# Subglacial meltwater routes of the Fennoscandian Ice Sheet

**DOI:** 10.1080/17445647.2022.2071648

**Published:** 2022-05-13

**Authors:** Nico Dewald, Stephen J. Livingstone, Chris D. Clark

**Affiliations:** Department of Geography, The University of Sheffield, Sheffield, UK

**Keywords:** Glacial geomorphology, subglacial meltwater landforms, drainage network, Fennoscandian Ice Sheet, digital elevation model

## Abstract

Subglacial drainage systems are crucial elements of glaciers and ice sheets because they modulate ice flow velocity. However, logistical challenges of measuring subglacial processes beneath contemporary ice and natural limitations in long-term monitoring hinder our understanding about their spatio-temporal evolution. Subglacial meltwater landforms created by palaeo-ice sheets are records of past subglacial drainage systems and offer the potential to study their large-scale development throughout deglaciation. Although collectively recording subglacial drainage, individual meltwater landforms such as eskers, meltwater channels and meltwater corridors, which comprise tunnel valleys and meltwater tracks (assemblages of landforms in broad, elongated paths with irregular surface texture), have mostly been investigated as separate entities. Using high-resolution (1–2 m) digital elevation models, we map integrated networks of subglacial meltwater landforms, herein called subglacial meltwater routes, on an ice-sheet scale in Fennoscandia. Our map provides a basis for future research on the long-term evolution of subglacial drainage networks and its effect on ice dynamics of the Fennoscandian Ice Sheet.

## 1. Introduction

The flow dynamics of glaciers and ice sheets are modulated by basal meltwater, however, studying contemporary subglacial drainage systems directly remains challenging. Subglacial meltwater landforms created during past glaciations are powerful archives that can help to inform our understanding about the subglacial hydrology of modern glaciers and ice sheets (Greenwood et al., 2016). In recent years, the study of landforms has been facilitated by the advent of national-scale and freely available high resolution (<5 m) elevation data. These new data allow us to examine the morphology of landforms in unprecedented detail (Chandler et al., 2018; Dowling et al., 2013; Johnson et al., 2015).

Subglacial melt water drainage is frequently idealised into two endmember configurations: distributed flow at the ice-bed interface and diffusing through sedimentary beds, or with water mainly channelised into discrete threads. While there is an ongoing debate about the extent of distributed drainage configurations recorded in the landscape (Greenwood et al., 2016; Mäkinen et al., 2017), the record of discrete or channelised drainage, such as esker ridges (De Geer, 1897; Hummel, 1874) and meltwater channels cut into the bed (Sissons, 1960, 1961), have long been recognised.

Esker ridges consist of glaciofluvial sediment typically deposited within subglacial conduits carved up into the ice (Röthlisberger, 1972). They can form extensive networks over previously glaciated areas and both their frequent superposition on top of other glacial landforms and numerical modelling suggests deposition close to the ice margin during deglaciation (e.g. Beaud et al., 2018; Hewitt & Creyts, 2019; Kleman & Borgström, 1996; Lewington et al., 2020; Livingstone et al., 2015; Storrar et al., 2014). Because the hydraulic potential below ice sheets is mostly controlled by the ice surface slope, which typically follows the direction of the ice flow, subglacial meltwater is broadly forced towards the ice margin in a similar orientation as the ice flow itself. Thus, the orientation of esker ridges allows the reconstruction of ice flow directions close to palaeo-ice margins (Stroeven et al., 2016). Eskers observed on palaeo-ice sheet beds rarely seem to be older than the most recent glaciation (cf. Storrar et al., 2014; Stroeven et al., 2016) although some exceptions have been found to be preserved from earlier stages (Johansson & Kujansuu, 1995; Lagerbäck & Robertsson, 1988; Sutinen & Middleton, 2021).

Meltwater channels, sometimes called Nye-channels, are cut into sedimentary or bedrock substrates and are typically <10 m deep and 10–20 m in width (Grau Galofre et al., 2018). Larger channels up to several kilometres wide, 10s kilometres long and 10s to 100s metres deep are called tunnel valleys (Kehew et al., 2012; Kirkham et al., 2021; van der Vegt et al., 2012). The term tunnel channel has been applied to describe large valleys interpreted as fully occupied by water during their formation (Clayton et al., 1999). Here, we will use the term tunnel valley in its widest sense, to refer to both tunnel valleys and tunnel channels (Livingstone & Clark, 2016). Subglacial meltwater channels and especially tunnel valleys were potentially reused by meltwater during the course of one or several glaciations (e.g. Atkinson et al., 2013; Jørgensen & Sandersen, 2006; Kirkham et al., 2020; Piotrowski, 1994).

It has recently been recognised that the repertoire of subglacial meltwater landforms described above is often associated with wide (100s m to km) meltwater tracks that are interpreted to be transitory spatial extensions of the channelised drainage, which are hydraulically connected to parts of the distributed drainage system (Lewington et al., 2020). Meltwater tracks are broad, regularly spaced traces of subglacial fluvial activity that frequently contain eskers, washed bedrock surfaces and/or hummocks (e.g. Folinsbee, 1952; St-Onge, 1984; Wilson, 1939, 1945). Such features have been reported in a number of studies from different locations and have variously been termed ‘meltwater corridors’, ‘hummock corridors’, ‘erosional corridors’ or ‘washed zones’ (Burke et al., 2011; Peterson et al., 2017; Rampton, 2000; Sharpe et al., 2017; Ward et al., 1997). More recently, associations of triangular to chevron-shaped landforms and elongated escarpments, broadly called murtoos and murtoo-related landforms, have been identified within meltwater tracks in both Finland and Sweden (Ahokangas et al., 2021; Mäkinen et al., 2017; Ojala et al., 2021; Peterson et al., 2017). They are thought to have formed by a range of subglacial processes associated with increased or fluctuating delivery of meltwater to the ice sheet bed (Ojala et al., 2021; Peterson Becher & Johnson, 2021).

The geomorphological literature has often investigated the various types of subglacial meltwater landforms in isolation (i.e. focussing on either eskers, meltwater channels, meltwater tracks, etc.). However, observations at various scales indicate that they do not form independently of one another, but each are smaller building blocks of a wider drainage network (Ahokangas et al., 2021; Boulton et al., 2009; Hebrand & Åmark, 1989; Jørgensen & Sandersen, 2006; Lewington et al., 2020; Salamon & Mendecki, 2021; Storrar & Livingstone, 2017). Accordingly, Lewington et al. (2020) applied the term ‘meltwater corridor’ as a superordinate category including tunnel valleys and meltwater tracks (Table 1). This categorisation is based on their frequent associations, transitions to and from esker ridges, and their similar morphometric characteristics (cf. Jørgensen & Sandersen, 2006; Peterson et al., 2018). Here we follow the idea of perceiving meltwater landforms collectively to learn more about the hydrological networks beneath palaeo-ice sheets. Our focus is on the Fennoscandian Ice Sheet and in this study, we present an ice sheet-scale map of subglacial meltwater routes.

**Table 1.** Classification hierarchy of subglacial meltwater landforms used in this study adapted from Lewington et al. (2020).

Subglacial Meltwater Route			
Esker	Subglacial Meltwater Channel	Subglacial Meltwater Corridor	
		Tunnel Valley	Subglacial Meltwater Track

Note: Subglacial meltwater route is the overarching term that refers to all subglacial meltwater landforms. Both tunnel valleys and subglacial meltwater tracks are herein referred to as subglacial meltwater corridors. Subglacial meltwater tracks include features referred to as ‘hummock corridor’ (Peterson & Johnson, 2018), ‘glaciofluvial corridor’ (St-Onge, 1984), ‘washed zones’ (Ward et al., 1997) and ‘erosional corridor’ (Burke et al., 2011). References are selected examples and not exhaustive. The term ‘tunnel valley’ is used in its widest sense (Livingstone & Clark, 2016).

### 1.1. Regional setting and previous work

The Fennoscandian Ice Sheet (FIS) formed the largest ice mass of the Eurasian Ice Sheet complex that repeatedly covered northern Europe during the Quaternary (Batchelor et al., 2019; Hughes et al., 2016). At its last maximum extent around 21–20 ka BP, the FIS covered the Scandinavian Peninsula, parts of Denmark, northern Germany and Poland to the South and reached several 100s km into western Russia to the East (Hughes et al., 2016; Stroeven et al., 2016). Following its peak in areal extent, the ice sheet retreated towards the Scandes mountain range where it disintegrated around 10 ka BP (Hughes et al., 2016; Stroeven et al., 2016). The Scandes mountain range in western Fennoscandia is the dominant topographic feature and reaches elevations of up to more than 2400 m a.s.l. Towards the East, the Fennoscandian topography becomes less variable and elevations >1000 m a.s.l. are rarely reached. Typical elevation values outside of the Scandes mountain range vary between 50–400 m a.s.l. The bulk of the geologic basement in Fennoscandia consists of Precambrian and Caledonian crystalline rocks with minor occurrences of sedimentary bedrock (Koistinen et al., 2001; Lahtinen, 2012). During its retreat, the ice sheet left widespread evidence of subglacial meltwater flow including extensive esker networks (Stroeven et al., 2016), meltwater tracks comprising hummocky topography and murtoos (Ahokangas et al., 2021; Peterson et al., 2017), tunnel valleys (Jørgensen & Sandersen, 2006; Peterson et al., 2018; van der Vegt et al., 2012) and inner gorges attributed to channelised subglacial meltwater erosion (Jansen et al., 2014).

There is a substantial literature dealing with the variety of subglacial meltwater features on both local and regional scales (e.g. Dahlgren, 2013; De Geer, 1910, 1940; Hättestrand, 1998; Hättestrand & Clark, 2006; Hebrand & Åmark, 1989; Jansen et al., 2014; Johansson, 2003; Johansson & Kujansuu, 1995; Lundqvist, 1997; Öhrling et al., 2020; Peterson et al., 2017, 2018; Peterson & Johnson, 2018). However, with a few exceptions (e.g. Shackleton et al., 2018), previous studies capturing subglacial drainage at the ice sheet scale in Fennoscandia have mainly targeted esker ridges (e.g. Boulton et al., 2001, 2009; Clark & Walder, 1994; Kleman et al., 1997; Stroeven et al., 2016). Previously published esker maps depict extensive arrays of esker ridges spreading from the Scandes mountain range across Fennoscandia. Spatially variable ridge topologies allow the identification of individual ice lobes across Finland and reveal changing palaeo-flow directions in northern Fennoscandia (Boulton et al., 2009; Punkari, 1994, 1997; Stroeven et al., 2016). In a recent study, Ahokangas et al. (2021) mapped subglacial meltwater routes in Finland and found them to form extensions to known esker pathways, revealing a wider network of former subglacial meltwater paths.

## 2. Methodology

### 2.1. Data

**Figure 1.**
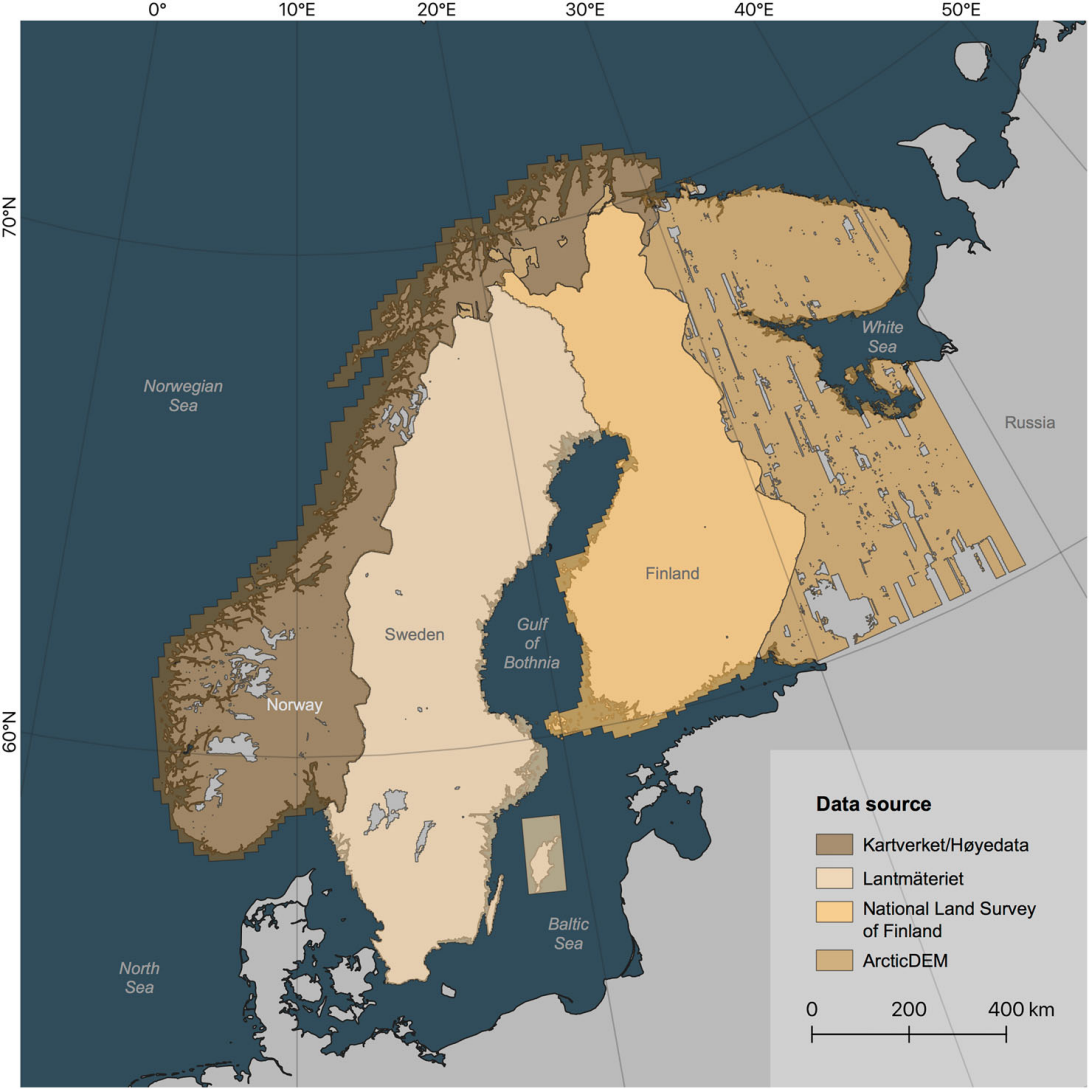
Data source and coverage for high-resolution elevation data used in this study. Mapping was restricted to indicated onshore areas. Grey onshore areas have not been mapped. Note larger gaps in Norway and Russian Karelia. Also note that ArcticDEM is a DSM comprising vegetation, whereas LiDAR-derived DEMs for Norway, Sweden and Finland are DTMs that show the bare earth surface. Offshore areas (darker shades for each colour) are not considered in this study.

For our mapping, we used high-resolution (1–2 m) national digital elevation models (DEMs; e.g. Dowling et al., 2013) of Norway, Sweden and Finland, and the ArcticDEM (2 m; Porter et al., 2018) for Russian Karelia and small areas in Norway (Figure 1). We used ArcMap 10.7 to create hillshaded digital elevation models with 45° altitude and contrasting azimuths of 45° and 315° to capture azimuth-parallel features (Chandler et al., 2018). The spatial coverage of our data varies and is dependent on the respective dataset i.e. country. While Sweden and Finland have good coverage overall, the data for Norway and Russia includes larger gaps (Figure 1). The ArcticDEM is a digital surface model (DSM) generated by stereo processing of satellite imagery (Porter et al., 2018) whereas national topographic datasets used in our study comprise LiDAR-derived digital terrain models (DTMs; Dowling et al., 2013). Because DSMs include tree canopies they tend to be less suitable for landform detection in forested regions such as Fennoscandia.

### 2.2. Landform descriptions

#### 2.2.1. Eskers

Eskers are straight to sinuous ridges (Figure 2 A–C) mostly consisting of glaciofluvial sand and gravel which can be traced over 10s of kilometres. They are often expressed as linear ridges but can also comprise beads, fans and enlargements (e.g. De Geer, 1897, 1910, 1940; Dewald et al., 2021; Gorrell & Shaw, 1991; Hebrand & Åmark, 1989; Lindström, 1993; Livingstone et al., 2019; Persson, 1974; Storrar et al., 2015, 2020). Single ridges are the most common type and are typically several 10s m wide and metres to 10s m high. In some places, multi-ridged esker segments can be observed (Figure 2G). Esker beads are generally rare in our study area, however, where present, they tend to be associated with De Geer moraines (cf. De Geer, 1897, 1910, 1940; Livingstone et al., 2019). Esker enlargements are spatially confined ridge widenings (typically <1.5 km in length) that can be found across Fennoscandia (Dewald et al., 2021; Lindström, 1993). Figure 2.Examples of subglacial meltwater landforms in Fennoscandia. **A**: Esker ridge in a meltwater track. Note characteristic small-scale irregularities of the meltwater track and continuation of the latter after the esker ridge disappears. Also note ribs towards the bottom of the image. **B**: A gap between esker ridge segments characterised by irregular topography. **C**: Esker ridges with lateral splay. **D**: Meltwater track with crenulated edges. Note that the corridor outline has been partially removed to improve visibility of features. Also note the track of ribs towards the bottom right. **E**: Irregular topography of meltwater track in otherwise smooth surface. Note irregular topography behind the ridge towards the right. **F**: Esker and fan complex. Note superficial channels and multi-ridged esker segments close to the fan apex. **G**: Multi-ridged esker segment. Note adjacent irregular topography. **H**: Esker ridge in valley (meltwater corridor) and confluent meltwater track without esker (indicated by dashed line). **I**: Esker ridge and continuation of meltwater route via a narrow channel (indicated as tunnel valley). Note that esker ridge is surrounded by irregular terrain, likely indicating a continuation of the indicated tunnel valley. **J**: Meltwater track in smooth terrain. Note embayment of moraine ridges and esker ridge on the right. **K**: Irregular terrain track in bedrock area. Note small-scale irregularities inside meltwater track margins. **L**: Confluent valleys (meltwater tracks) comprising hummocks, ribs and murtoos*. Note lineations and partially irregular terrain in areas between valleys. **M**: Association of esker ridges and murtoos* with ribbed moraine tracks. Note confluence of moraine tracks and smooth surface in between. **N**: Tunnel valley with crenulated edges cutting through a hill (∼20 m high). **O**: Complex zone of valleys comprising murtoos* and esker ridges. Note flow direction and increasing valley size towards southeast. **All panels**: Brown colours denote higher elevations. White arrows denote meltwater downflow direction. *refers to murtoos and murtoo-related subglacial landforms (Ojala et al., 2021). Data sources: A, C-E, G-O: GSD-Höjddata, grid 2+ © Lantmäteriet. B contains data from National Land Survey of Finland Elevation model 2 m 11/19. F: ArcticDEM, 2 m.A collage of hillshaded digital elevation models showing examples of subglacial meltwater landforms.

**Figure:**
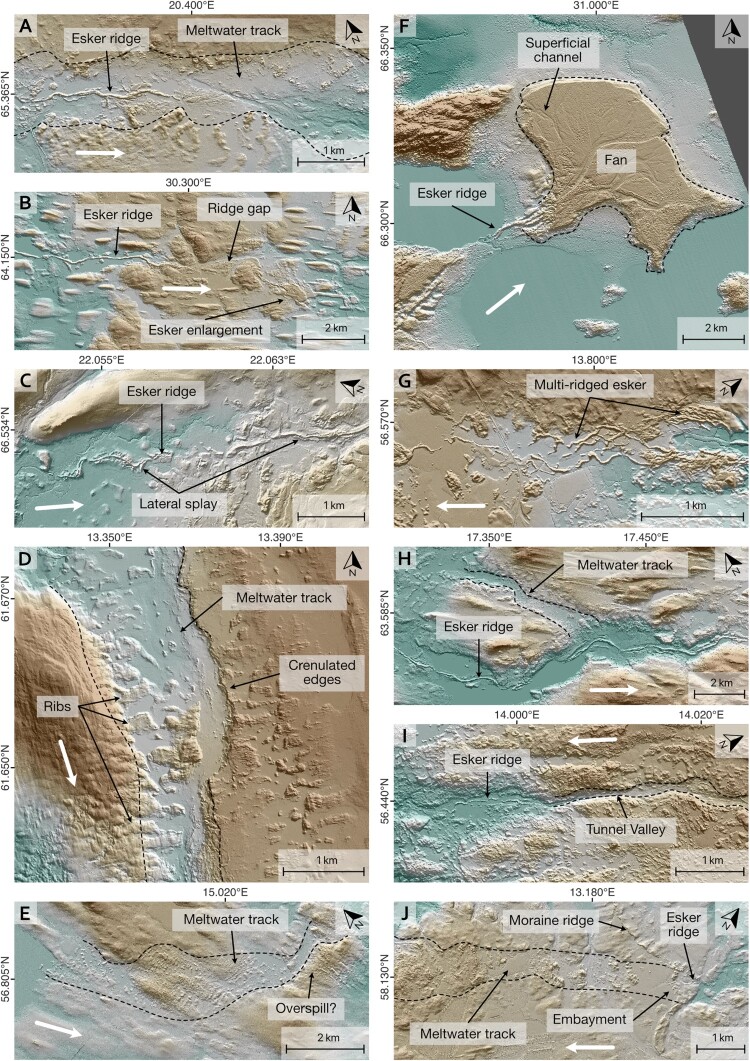

**Figure:**
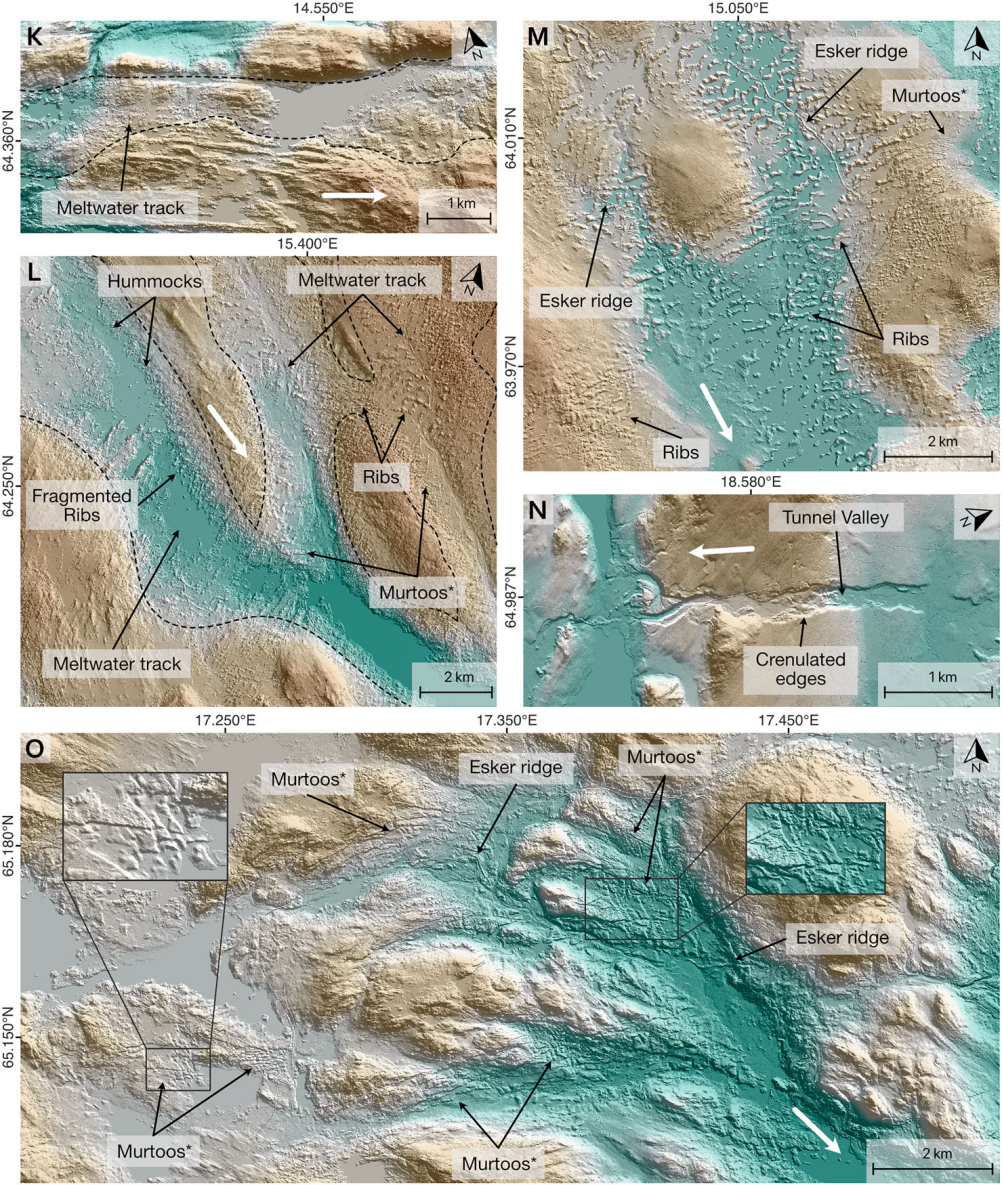

**Figure 3.**
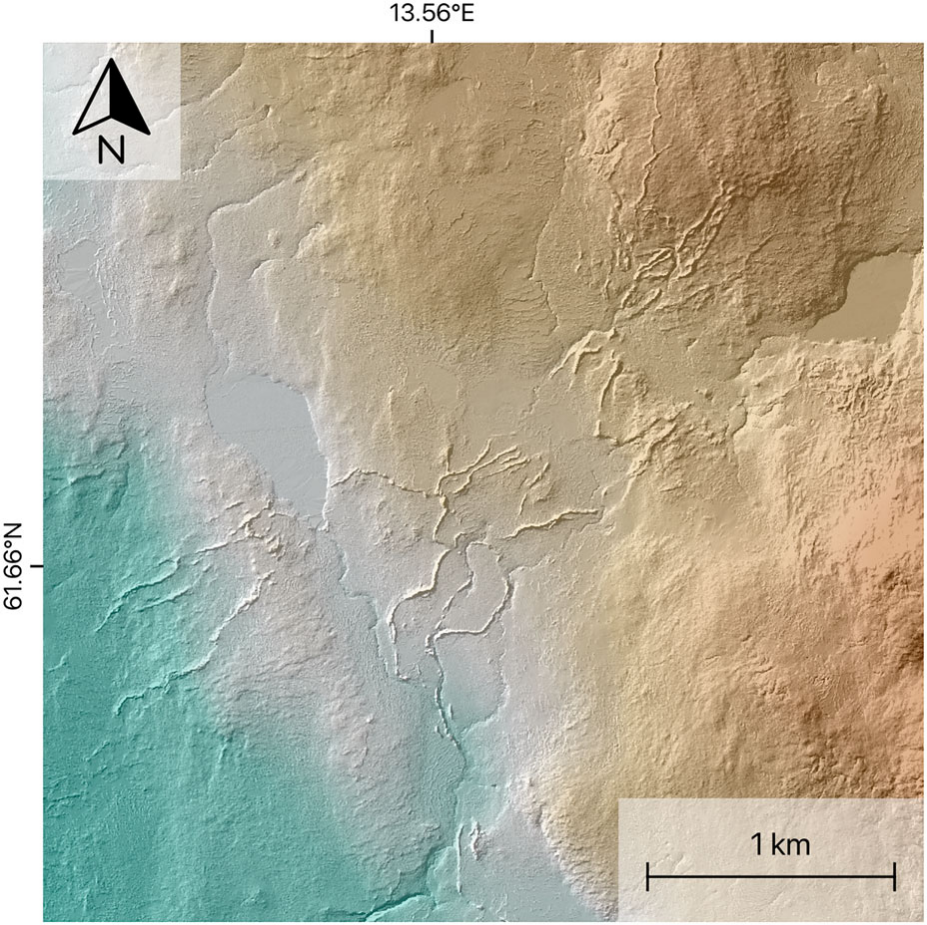
Array of esker-like ridges with variable orientations and no apparent relationship to other meltwater features. Since these complex geometries are rather uncommon for eskers in our study area we refrained from including them on the map to prevent potential bias. Data source: GSD-Höjddata, grid 2+ © Lantmäteriet.

We also note arrays of complexly arranged esker-like ridges with highly variable orientations and frequent cross-cuttings (Figure 3), some of which seem to be related to meltwater corridors. Where this is the case, they are interpreted to have formed by subglacial fluvial processes. In other cases, where a relationship to other meltwater features was not evident (Figure 3), they were not added to the map.

#### 2.2.2. Subglacial meltwater corridors

*Tunnel valleys* are elongated depressions cut into sediment or bedrock (Figure 2I, N). They are characterised by their orientation broadly parallel or concordant with former ice flow, undulating thalwegs, abrupt (dis-)appearances (Kehew et al., 2012; Kirkham et al., 2020, 2021; van der Vegt et al., 2012) and association with other subglacial meltwater landforms such as esker ridges (e.g. Jørgensen & Sandersen, 2006; Kirkham et al., 2021; Salamon & Mendecki, 2021). The cross-sectional morphology of tunnel valleys can vary between both U and V-shaped, reflecting different degrees of glacial and subglacial fluvial erosion, respectively (Jansen et al., 2014; Jørgensen & Sandersen, 2006; van der Vegt et al., 2012).

*Meltwater tracks* are linear zones of subglacial fluvial activity often characterised by coarse textures that frequently contrast with surrounding, usually smoother, surfaces (e.g. Figure 2E, J–M). Observable structures within meltwater tracks include irregular hummocks (Figure 2J–L; Peterson et al., 2017), murtoos and murtoo related landforms (Figure 2L–M, O; Ahokangas et al., 2021; Ojala et al., 2019, 2021), ribs and transverse ridges (Figure 2D–E, L–M; Peterson et al., 2017), subglacial meltwater channels (cf. Vérité et al., 2021), and scoured bedrock surfaces (Figure 2K; Rampton, 2000; Sharpe et al., 2017; St-Onge, 1984). Combinations of these can occur within a single meltwater track (e.g. Figure 2D–E, L). Meltwater tracks can be incised into the bed or manifest as positive relief (Peterson & Johnson, 2018). Where meltwater tracks are cut into sediment, their margins occasionally exhibit crenulated (or scalloped) edge morphologies (Figure 2D, N; Lewington et al., 2020; Rampton, 2000).

The dimensions of structures within meltwater tracks range from metres to 10s metres for irregular hummocks and transverse ridges and up to 10s to 100s metres for murtoo and murtoo related landforms, ribs and crenulated edges (cf. Ojala et al., 2021). Where meltwater tracks are associated with ribs, they can be fragmented or transition to murtoos and murtoo related landforms (e.g. Figure 2L–M, cf. Vérité et al., 2021). Both ribs and transverse ridges can be oblique to the corridor axis.

#### 2.2.3. Subglacial meltwater channels

**Figure 4.**
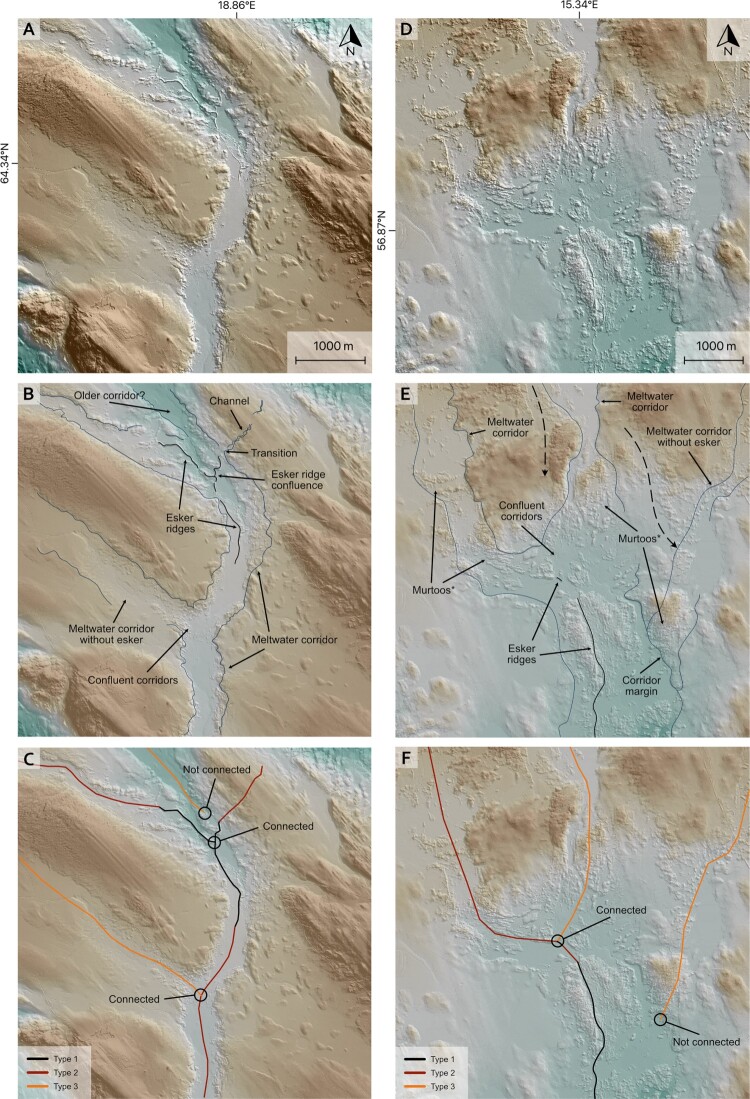
Original DEM, interpretation and concluded mapping for two example locations (**A**-**C** and **D**-**F**). Subglacial meltwater routes are combinations of different meltwater signatures (**A**/**D**) that line up and form a coherent drainage pathway (**B**/**E**). Where geomorphological evidence does not indicate a direct connection (**B**/**E**), the respective routes are not connected (**C**/**F**). Also note minor potential meltwater tracks in E (dashed arrows). *refers to murtoos and murtoo related subglacial landforms (Ojala et al., 2021). Data source: GSD-Höjddata, grid 2+ © Lantmäteriet.

Subglacial meltwater channels are m-scale erosional troughs formed by channelised meltwater flow at the ice sheet bed. They typically have undulating long-profiles, high sinuosities and do not follow local topographic gradients (Grau Galofre et al., 2018; Greenwood et al., 2007). They can be arranged in complex bifurcating and anastomosing networks but generally follow palaeo-hydraulic gradients (Grau Galofre et al., 2018; Greenwood et al., 2007). Due to their relatively small dimensions, subglacial meltwater channels were rarely mapped individually using our generalised mapping approach. Instead we often observed them in association with subglacial meltwater corridors and eskers (Figure 2O). In the relatively rare case of esker-channel transitions, subglacial meltwater channels were treated as meltwater corridors (Figure 4).

### 2.3. Mapping approach

To ensure a systematic mapping approach, we created sets of east–west trending lines with a north–south spacing of 12 km for each country. These were used as guidelines along which our mapping was orientated. All features were mapped at scales between 1:20,000 and 1:40,000 in ArcMap 10.7 or QGIS 3.

The large-scale nature of our study (1.4 × 10^6^ km^2^) required making compromises regarding the detail and arrangement of our mapping. We adapted the approach introduced by Lewington et al. (2020) in combining different records of subglacial drainage into a common signature termed ‘subglacial meltwater route’ (Table 1). This has the advantage of capturing different landforms that are likely to record similar phenomena (e.g. Boulton et al., 2009; Jørgensen & Sandersen, 2006; Salamon & Mendecki, 2021) and provides a way to map large areas within reasonable time constraints while still capturing details on hydrological routes. We classified subglacial meltwater route segments based on the respective landforms and their association with each other. We distinguish three types of subglacial meltwater routes: (type 1) esker ridges, (type 2) meltwater corridors associated with esker ridges and (type 3) additional meltwater corridors.

*Esker ridges* (type 1) have been mapped along their crestlines (Figure 4). Where esker ridges transition into meltwater corridors the respective corridor was mapped as a *meltwater corridor associated with esker ridge* (type 2). In cases where esker ridges occur *within* a meltwater corridor (Figure 2A), the respective esker ridge has been mapped (Figure 4). Features classified as *additional meltwater corridors* (type 3) either show a decline in their morphological distinctiveness compared to associated subglacial meltwater routes or have no direct association to esker ridges (Figure 4). The two configurations can be distinguished by their (non-)association to other subglacial meltwater routes on the map.

## 3. Results and discussion

**Figure 5.**
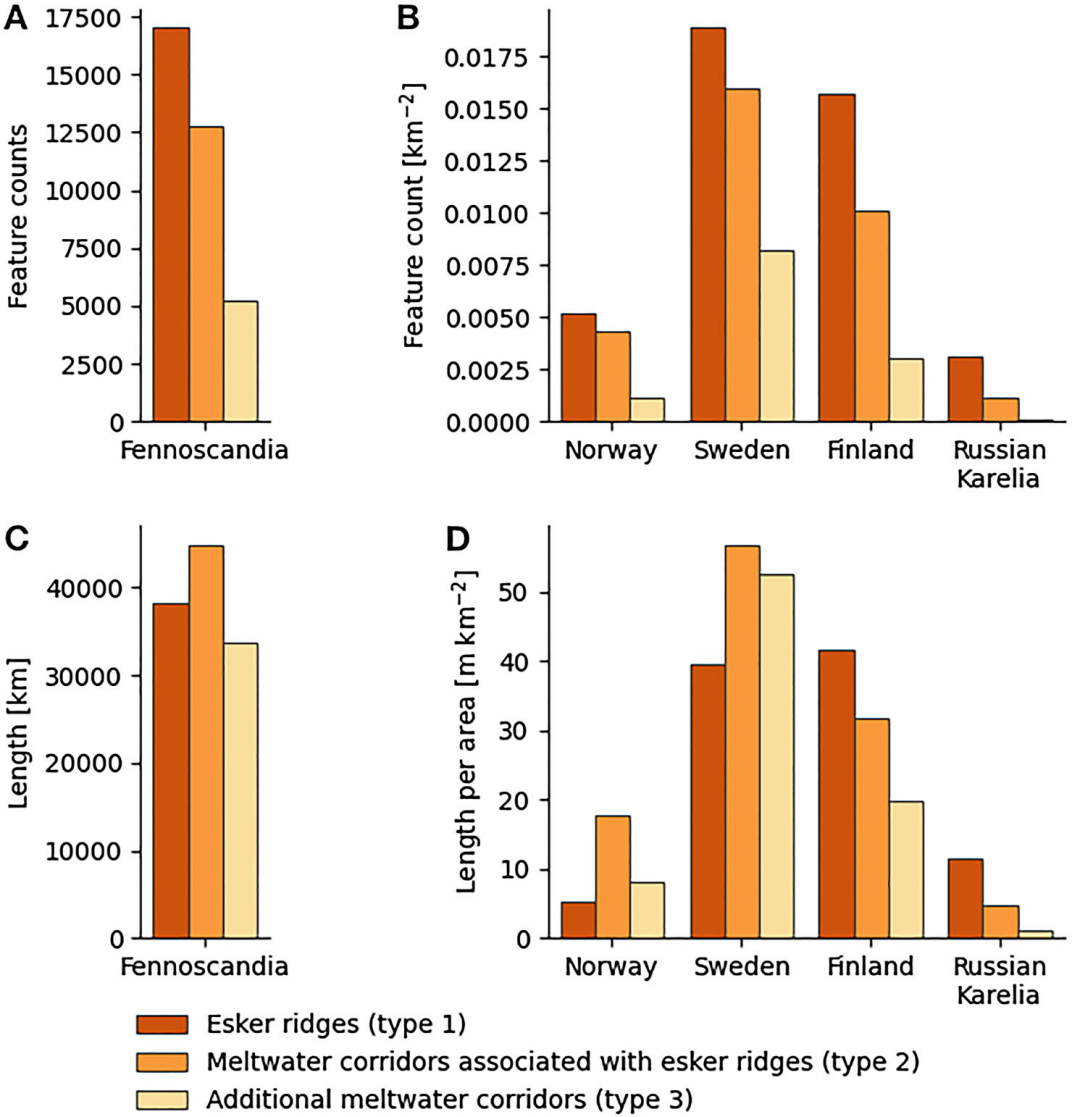
Mapped feature statistics by type of meltwater route. Type 1: Esker ridges; type 2: meltwater corridors associated with esker ridges; type 3: additional meltwater corridors. **A**: Total mapped feature counts across study area. **B**: Normalised feature counts for individual countries. **C**: Sum length of mapped features across study area. **D**: Normalised feature lengths for individual countries. Note that differences in data quality likely skewed the present statistics.

In concert with journal guidelines, our analysis is kept to a minimum and will be the subject of future work. We mapped a total of 34,951 individual subglacial meltwater route segments with a combined length of 117,188 km (Figure 5A&C). Overall, type 1 features make up 38,263 km (32.7%), type 2 features make up 44,932 km (38.3%) and type 3 features make up 33,993 km (29.0%) (Figure 5C). Type 1 features show the highest number of mapped features in every country (Figure 5B). However, the total length of mapped type 2 features is higher than the total length of mapped type 1 features (Figure 5C) reflecting the generally shorter lengths of mapped esker ridges. Since feature counts depend on choices made by the respective mapper, length values likely provide a better representation of these data.

Total subglacial meltwater route length and length density are highest in Sweden and Finland (Figure 5B&D). In contrast, subglacial meltwater routes are less abundant and esker ridges are typically smaller and/or more fragmented in Norway. The general distribution of esker ridges (type 1) and subglacial meltwater corridors (type 2 and 3) is slightly skewed with type 1 being the predominant landform in large parts of Finland and Russian Karelia, while type 2 and 3 show higher densities in both Norway and Sweden (Figure 5D). However, values for Norway and Russian Karelia are likely affected by differences in data coverage and DTM availability (Figure 1).

### 3.1. Drainage network pattern

The ice sheet-wide distribution of subglacial meltwater routes is consistent with previous large-scale esker maps (Boulton et al., 2009; Stroeven et al., 2016). However, the extended array of meltwater landform types and higher resolution of mapping yields much denser networks and spatial variations become more pronounced (cf. Ahokangas et al., 2021; Lewington et al., 2020). At an ice sheet-scale, subglacial meltwater routes spread from the Scandes mountain range predominantly towards the southeast across Fennoscandia (see main map). Notable exceptions can be found in northern Fennoscandia where meltwater routes are directed towards the northeast and in southern Sweden where they are partly NE-SW orientated.

Areas with apparent cross-cutting between subglacial meltwater routes can be found in northern Sweden and central Finland (see main map) where cold-based conditions have been inferred from earlier studies (e.g. Kleman, 1994; Kleman & Hättestrand, 1999; Sarala, 2005). Additionally minor apparent cross-cutting can be found in west-central Sweden (cf. Kleman et al., 1992). The SW-NE trending subglacial meltwater routes in northern Fennoscandia are associated with a decrease in subglacial meltwater route density to the southeast, associated with an inferred palaeo-ice divide position (Johansson et al., 2011 and references therein). The Kola Peninsula itself is largely absent of any subglacial meltwater routes consistent with inferred cold-based conditions (cf. Boyes et al., 2021; Hättestrand & Clark, 2006).

**Figure 6.**
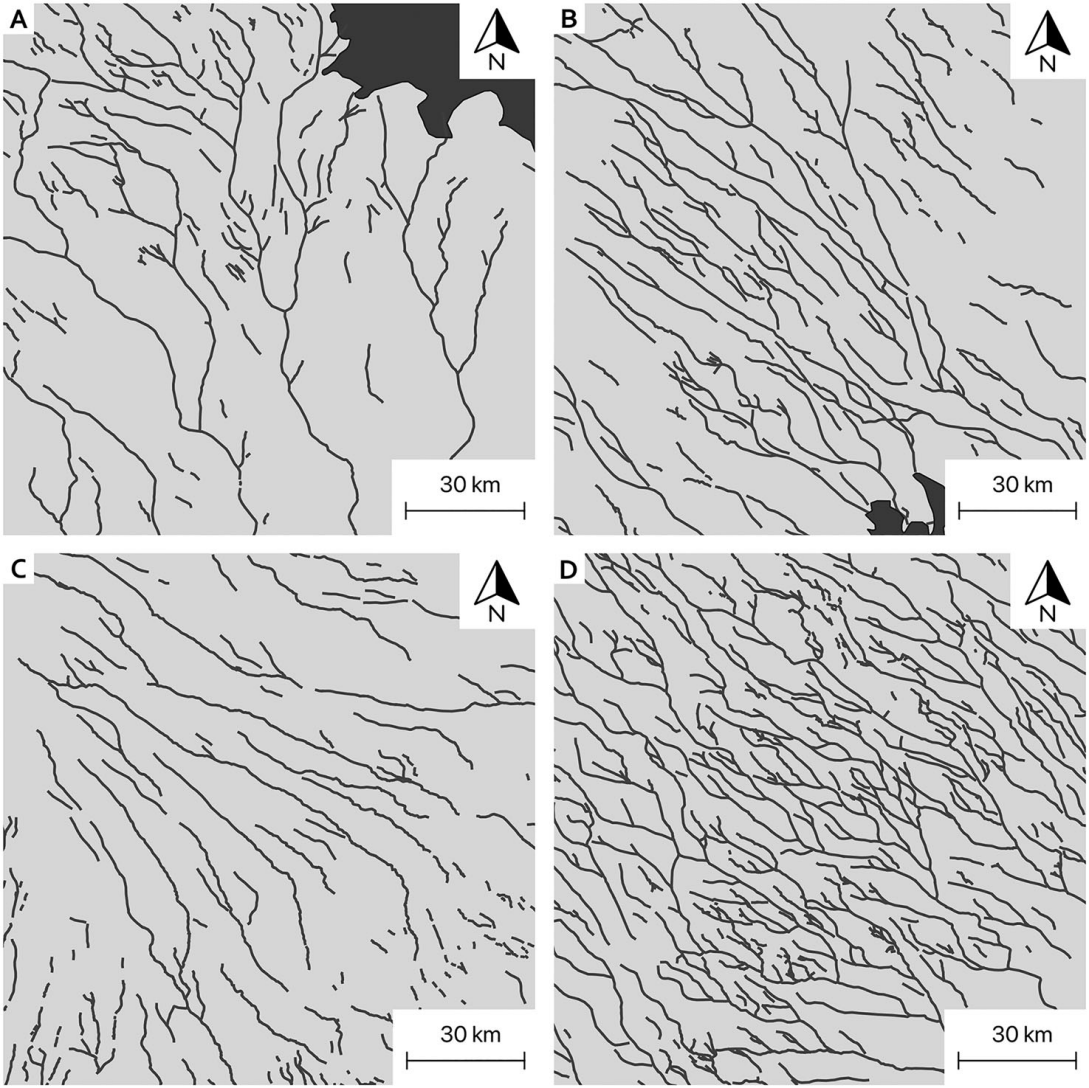
Subglacial meltwater drainage network pattern. Note equal scale for all panels. **A**. Large-scale dendritic pattern NW of Stockholm. **B**: Parallel pattern in northern Sweden. **C**: Diverging pattern in SE Finland. **D**: Complex anastomosing pattern in central Sweden. Note apparent bandings from NE towards SW in both C and D. **All panels**: palaeo-ice flow towards SE.

We identify a variety of drainage patterns in mapped subglacial meltwater routes (Figure 6). As observed in previous studies (Boulton et al., 2009; Punkari, 1980, 1994, 1997; Stroeven et al., 2016), glaciodynamic control on subglacial drainage is evident from distinct lobe structures comprising a blend of both divergent (Figure 6C) and convergent zones across Finland and southern Sweden. In Sweden, the character of subglacial drainage patterns is generally more distributed, comprising both dendritic (Figure 6A), parallel (Figure 6B) and complex anastomosing patterns (Figure 6D). The dendritic pattern formed by esker ridges in east-central Sweden (Figure 6A, east Svealand) is accompanied by an overall decrease in feature density. In contrast, areas in southern and northern-central Sweden, and in the Salpausselkä area in Finland have relatively high feature densities. Mountainous regions with more complex topographies in Norway and western Sweden tend to show irregular and fragmented drainage patterns (see main map).

### 3.2. Comparison to previous work

We compared our mapping to previous studies that documented subglacial meltwater features, each varying with respect to detail and/or mapped area. For this, we used a comprehensive and detailed database of glacial landforms in Finland (Putkinen et al., 2017), integrated maps of subglacial meltwater traces across Finland (Ahokangas et al., 2021) and northern Sweden (Jansen et al., 2014), and detailed glacial geomorphological mapping of the South Swedish Uplands (Peterson et al., 2017).

**Figure 7.**
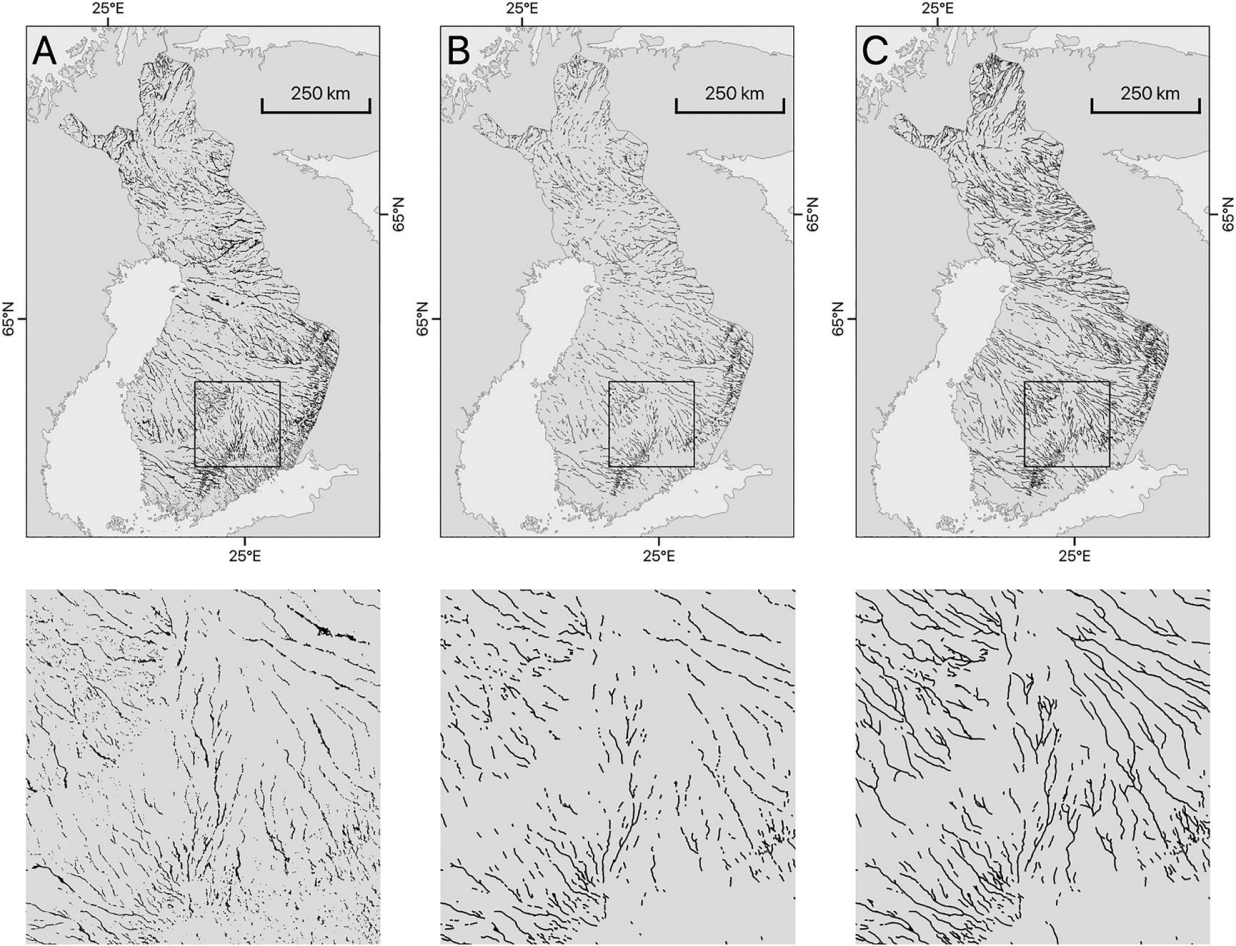
Comparison of mapped meltwater features in Finland (**A**, Putkinen et al., 2017) to mapping presented in this study (**B**, esker ridges; **C** all meltwater routes). Boxes in A, B and C denote identical areas shown beneath each panel. Note that large-scale drainage geometries and changes in feature density are mostly equivalent. Also note that detailed mapping in A captures size differences of meltwater features while our data does not.

A comparison to eskers extracted from a LiDAR-derived database of glacial landforms in Finland (Putkinen et al., 2017) indicates equivalent changes in drainage patterns and feature densities (Figure 7). Larger deviations occur around the south and south-west coast of Finland where our map shows lower feature densities, likely because of the indistinct landform morphology caused by marine and lacustrine inundation during deglaciation. Despite the qualitatively similar large-scale pattern, our esker data (type 1) show substantially lower feature counts (ca. 87%, cf. Figure 7) as would be expected due to the generalisations imposed by our approach and the applied mapping scale. However, Ahokangas et al. (2021) provide additional statistics on esker ridge counts and lengths derived from this dataset, which are comparable to esker ridges in Finland from our study (Table 2). Visual comparison to subglacial meltwater routes in Finland (Ahokangas et al., 2021), yields qualitatively similar results (cf. Figure 4A in Ahokangas et al., 2021), however, a direct statistical comparison is difficult due to deviating classification systems.

**Table 2.** Comparison of feature counts (*N*) and length statistics (in km) of esker ridges (type 1) in this study with Ahokangas et al. (2021) and Peterson et al. (2017) for each study area.

	This study (type 1)	Ahokangas et al. (2021)
*N*	5294	6870
Min	0.003	0.04
Max	123.6	175.33
Median	1.3	1.28
Mean	2.64	3.94
1st Quartile	0.65	0.53
3rd Quartile	2.87	3.51
	This study (type 1)	Peterson et al. (2017)
*N*	1259	3532
Sum length	1453	1481

Note: As mentioned in Section 3, feature counts depend on choices made by the respective mapper and may deviate although the sum feature length is similar, as is the case here with Peterson et al. (2017) and this study.

**Figure 8.**
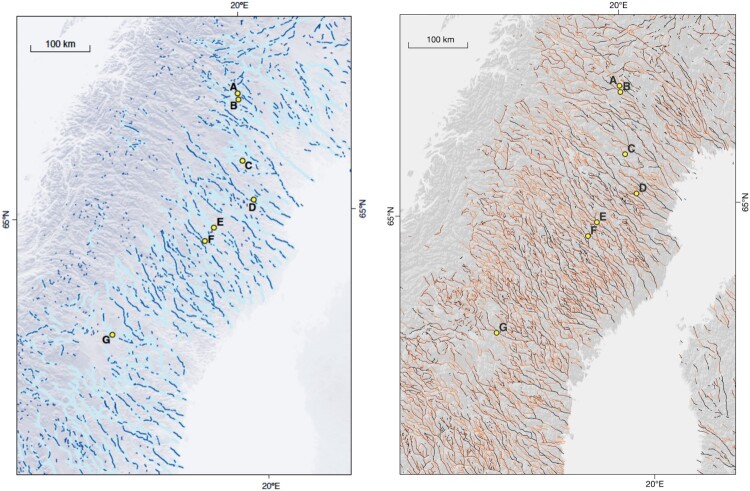
Comparison of integrated mapping by Jansen et al. (2014) to mapping presented in this study (right panel). Left panel shows ‘reconstructed subglacial drainage paths […] indicated (thick light-blue lines) by linking esker segments [dark blue lines] according to other signs of subglacial meltwater drainage, such as channels and glaciofluvial sediments of more diffuse morphology’. (Jansen et al., 2014; supplementary Fig. 16, reproduced with permission of Springer Nature). Yellow points indicate sample locations by Jansen et al. (2014) and are shown here for reference. Esker ridges after Hättestrand (1998). Right panel shows equivalent area of the map presented in this study. Yellow points are equivalent to left panel. Note the overall similar pattern and that both increases (e.g. area between G and D) and decreases (e.g. SW of G) in feature density are visible in both panels. Also note that higher elevated areas in B are dominated by type 3 subglacial meltwater routes due to a dearth of esker ridges.

By linking esker ridges to other subglacial meltwater features, Jansen et al. (2014) created a map of ‘subglacial meltwater paths’ for their study area in north-central Sweden and surrounding areas, broadly equivalent to a combination of type 1 and type 2 subglacial meltwater routes from this study. Comparison between the maps reveals similarities in both general network pattern and feature density (Figure 8). The addition of type 3 subglacial meltwater routes in this study resulted in more detailed and in some areas (e.g. Scandes mountain range) more abundant subglacial meltwater routes (Figure 8).

Qualitative comparison to comprehensive, LiDAR-based mapping projects across the South Swedish Uplands (Peterson et al., 2017; cf. Peterson & Johnson, 2018) indicates that our map captures the existing drainage pattern giving us confidence that despite some generalisation, the approach is able to capture >∼90% of the subglacial drainage elements. This is supported by almost identical sum lengths for esker ridges across one of the selected study areas (Table 2).

### 3.3. Comments and limitations

#### 3.3.1. Temporal aspects

Repeated periods of glacially induced erosion in Fennoscandia led to numerous incised valleys in which ice flow concentrated during subsequent glaciations (Jansen et al., 2014; Li et al., 2005). Thus, persistent topographical characteristics created by earlier glaciations likely influenced subglacial drainage paths during the last glaciation (cf. Hebrand & Åmark, 1989). This complicates assessments of the temporal development of subglacial meltwater landforms.

Our map is based around esker ridges and their relationship to meltwater corridors. However, this relationship does not attest to a contemporaneous formation. It is rather an expression of the idea that glacial landscapes are composite records (e.g. Greenwood et al., 2016) with some components sharing a genetic link, in this case, the subglacial drainage network. For example, while meltwater may have persistently drained along a drainage route, it is likely that the manifestation of specific meltwater landforms occurred during different stages of deglaciation.

Traditionally, eskers are associated with the most recent deglaciation (Storrar et al., 2014; Stroeven et al., 2016) because repeated proglacial fluvial and/or subsequent glacial erosion would have likely destroyed them (Storrar et al., 2020). However, in some areas of Fennoscandia cross-cutting relationships (Johansson & Kujansuu, 1995; see also Section 3.1), radiocarbon ages from associated kettle holes (Lagerbäck & Robertsson, 1988) and stratigraphical inferences (Sutinen & Middleton, 2021) indicate that some eskers formed prior to the last deglaciation. Although the majority of esker ridges in Fennoscandia are reasonably inferred to record subglacial drainage during the most recent deglaciation (cf. Stroeven et al., 2016), evidence of older esker systems will need careful treatment during future analyses.

#### 3.3.2. Further limitations

Besides challenges with inferring the temporal evolution of drainage, we point out additional limitations of our study:

1. Variations in feature density between areas of different data sources might be due to differences in the respective datasets, and not necessarily reflect an actual change in subglacial meltwater route count. As discussed above, this is mostly due to the inclusion of tree canopies in DSMs (i.e. ArcticDEM) which impedes the identification of glacial meltwater landforms in forested areas.
2. Because feature dimensions are not considered, the map may give the impression that all subglacial meltwater routes have evacuated equal volumes of water which is unlikely to be realistic (cf. Figure 7 and Palmu et al., 2021). Subglacial discharges are expected to have varied in both space and time yielding a complex evolution beyond the qualitative scope of this map.
3. Because post-depositional modification or a lack of morphological imprint may have led to gaps in the landform record (Storrar et al., 2014, 2020), this can only be a minimum map of subglacial meltwater routes in Fennoscandia. We also note that despite improved data quality, our comparisons show that we have unintentionally missed features during the mapping process (see section 3.2).
4. Our study focuses on the terrestrial parts of Norway, Sweden, Finland and Russian Karelia only. Absence of mapped landforms in the surrounding areas (e.g. in and south of the Baltic Sea) does not suggest that subglacial meltwater landforms do not exist in these areas (e.g. Greenwood et al., 2016, 2017).

## 4. Summary

We present the first ice sheet-scale map of subglacial meltwater routes in Fennoscandia. Subglacial meltwater routes comprise eskers and meltwater corridors, which together form extensive networks throughout the area. We distinguish between esker ridges, meltwater corridors that are associated with esker ridges and additional meltwater corridors.

This map provides a basis for ice sheet-scale analyses of subglacial drainage networks in Fennoscandia, with a particular challenge being to derive the time sequencing of the development of the hydrological network during deglaciation. This will be the subject of future work, and we plan to use the map to inform ice sheet modelling experiments.

## Data

The data that support the findings of this study are openly available in ORDA (Online research data) at 10.15131/shef.data.17086472.

## Software

Hillshaded DEMs were created using Esri ArcMap 10.7. All features were digitised as polylines using Esri ArcMap 10.7. and QGIS 3.10.

## Supplementary Material

TJOM_A_2071648_Supplementary material
