# Supplementary material for: Subglacial meltwater routes of the Fennoscandian Ice Sheet
Source: J Maps. 2022 May 13;18(2):382–96. doi: 10.1080/17445647.2022.2071648 (PMC9810041; doi:10.1080/17445647.2022.2071648)
Supplement: TJOM_A_2071648_Supplementary material [file TJOM_A_2071648_SM6691.pdf]

Subglacial Meltwater Routes of the Scandinavian Ice Sheet

Nico Dewald[1], Stephen J. Livingstone[1], Chris D. Clark[1]

[1]Department of Geography, University of Sheffield, Sheffield, United Kingdom

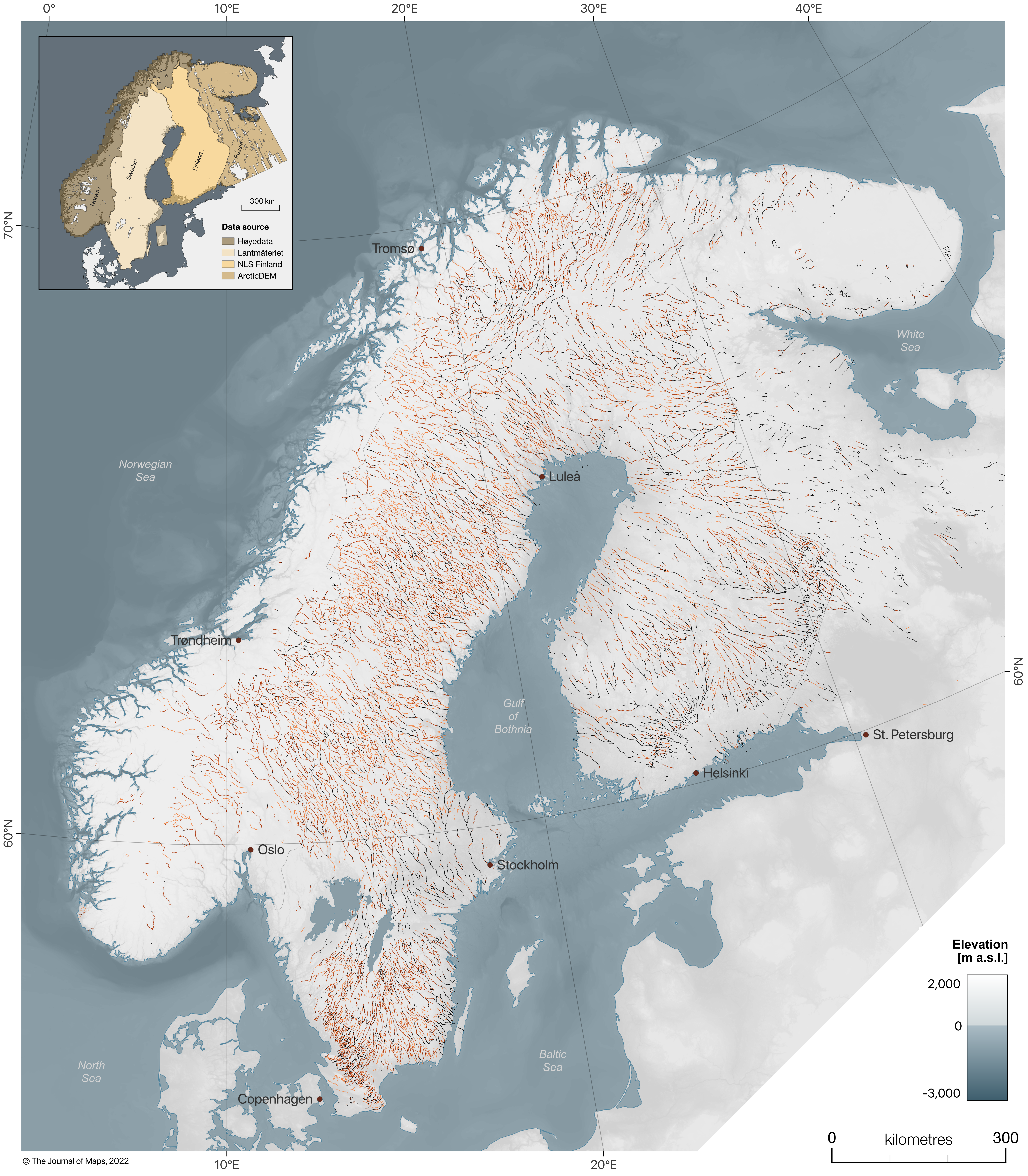

© The Journal of Maps, 2022

Elevation [m a.s.l.]

2,000

0

-3,000

0 kilometres 300

1:3,200,000
(on A1)

Subglacial Meltwater Routes

- Type 1 (esker ridges)
- Type 2 (meltwater corridors associated with esker ridges)
- Type 3 (additional meltwater corridors)

Projection & Datum
WGS 84 / North Pole Lambert Azimuthal Equal-Area
Europe (EPSG:3575)

Grid
Latitude & Longitude,
World Geodetic System 1984 (EPSG:4326), WGS 84

Elevation data
GEBCO Working Group (2021): GEBCO Grid 2021
